# Effect of Brazilian green propolis in chronic ulcer treatment: a randomized clinical trial

**DOI:** 10.1590/0034-7167-2023-0418

**Published:** 2024-09-06

**Authors:** Pascalle de Sousa Rocha, Luis Rafael Leite Sampaio, Gyllyandeson de Araújo Delmondes, Andreza Gysllaynny Delmondes Saraiva, Fábio Ferreira Perazzo, Fernando Luiz Affonso Fonseca, José Lucas de Souza, Luis Fernando Reis Macedo

**Affiliations:** I Faculdade de Medicina do ABC. Santo André, São Paulo, Brazil; II Universidade Federal de São Paulo. São Paulo, São Paulo, Brazil; III Instituto Federal da Paraíba. Sousa, Paraíba, Brazil; IV Universidade Regional do Cariri. Crato, Ceará, Brazil; V Universidade Federal do Vale do São Francisco. Petrolina, Pernambuco, Brazil; VI Faculdade de Medicina Estácio de Juazeiro do Norte. Juazeiro do Norte, Ceará, Brazil

**Keywords:** Ulcer, Wounds and Injuries, Biological Products, Clinical Trial, Enterostomal Therapy, Úlcera, Heridas y Lesiones, Productos Biológicos, Ensayo Clínico, Estomaterapia

## Abstract

**Objective:**

to assess the effectiveness of 5% Brazilian green propolis (ointment) in individuals with chronic ulcers.

**Methods:**

a randomized clinical trial, developed with 40 patients randomized equally to control group (treated with essential fatty acid) and experimental group (treated with 5% green propolis) for 30 days. The outcomes of interest were sociodemographic, clinical and laboratory characteristics, lesion characteristics, such as type of tissue in the bed, presence of exudate, edge characteristics, microbial content and pain.

**Results:**

regarding sociodemographic, clinical and laboratory characteristics, the two groups did not show statistically significant differences. After assessment in 30 days, an effect was observed for both treated groups, but for the experimental group, greater effectiveness in terms of the type of tissue in the bed, type of exudate, edge characteristics, microbial content and pain.

**Conclusion:**

propolis-based ointment showed a healing effect, presenting itself as a potential tool in healing chronic ulcers.

## RESUMEN

**Objetivo:**

evaluar la eficacia del propóleo (ungüento) verde brasileño al 5% en personas con úlceras crónicas.

**Métodos:**

ensayo clínico aleatorizado, desarrollado con 40 pacientes aleatorizados por igual al grupo control (tratados con ácidos grasos esenciales) y al grupo experimental (tratados con 5% de propóleo verde) durante 30 días. Los resultados de interés fueron características sociodemográficas, clínicas y de laboratorio, características de la lesión, tales como tipo de tejido en el lecho, presencia de exudado, características del borde, contenido microbiano y dolor.

**Resultados:**

en cuanto a las características sociodemográficas, clínicas y de laboratorio, los dos grupos no mostraron diferencias estadísticamente significativas. Luego de la evaluación en 30 días, se observó efecto para ambos grupos tratados, pero para el grupo experimental mayor efectividad en cuanto al tipo de tejido del lecho, tipo de exudado, características de los bordes, contenido microbiano y dolor.

**Conclusion:**

la pomada a base de propóleo mostró un efecto curativo, presentándose como una herramienta potencial en la curación de úlceras crónicas.

## INTRODUCTION

Chronic ulcers (CU) are a growing public health problem that affects millions of people worldwide. They can vary in severity and cause, often resulting from underlying problems such as circulatory/metabolic disorders or autoimmune conditions, which makes treatment much more challenging, significantly impacting the quality of life of those affected^(1)^.

Conditions associated with this cause lead to prolonged healing and increased costs for health services and patients. It is estimated that, in recent years, there has been a gradual increase in the incidence and prevalence of individuals with CU associated with chronic degenerative conditions. Therefore, recent studies indicate that the prevalence of CU of mixed etiology is 2.21 per 1,000 individuals^(2)^.

As a result, strategies are continually developed, such as technologies, new therapeutic approaches, extraction of materials and natural resources, among others, for CU treatment, enabling rapid action to meet patients’ needs and avoiding complications^(3)^. Therefore, the Brazilian National Policy on Integrative and Complementary Practices acts on therapeutic resource use, not derived from medicines, which seeks to recognize human care tradition, validating and including biological product use as a form of intervention for a range of health conditions^(4)^.

Therefore, Brazilian green propolis is a natural resource known for its chemical and pharmacological properties that can be beneficial in CU treatment. It is resin produced by bees from plant substances. However, it is important to note that propolis composition can vary depending on the geographic region and local plants from which bees obtain it^(5)^.

Brazilian propolis is rich in bioactive compounds that exhibit several therapeutic properties, such as antimicrobial, anti-inflammatory, antioxidant, analgesic properties, among others. It has been associated with accelerating the CU healing process, also stimulating collagen production, in addition to promoting angiogenesis^(6)^.

Thus, the importance of exploring the benefits of using Brazilian propolis for CU treatment is clear. Thus, the following question was addressed: will individuals with CU treated with 5% green propolis ointment (GPO) have a higher percentage of healing than those treated with conventional therapy?

## OBJECTIVE

To assess the effectiveness of using 5% GPO in treatment of individuals with CU.

## METHODS

### Ethical aspects

The intervention process and data collection occurred upon approval by the Research Ethics Committee (REC) and registration in the Brazilian Registry of Clinical Trials (ReBEC - *Registro Brasileiro de Ensaios Clínicos*) (RBR-294d68). All phases of the research strictly followed the ethical and legal requirements of Resolution 466/2012 of the Brazilian National Health Council/Ministry of Health. It is noteworthy that this study followed the necessary protocols to assist participants and their family members, respecting their autonomy and defending them in their vulnerability, by signing the Informed Consent Form, guaranteeing information confidentiality and freedom of participation in the group.

### Study design, place and period

This is a randomized controlled, single-blind clinical trial, developed in accordance with the CONsolidated Standards Of Reporting Trials (CONSORT)^(7)^ guidelines, carried out at a Nursing School Clinic in the city of Cajazeiras (06º53’24 “S; 38º33’ 43” W), Paraíba, Brazil. The study was carried out from March to October 2019.

### Population and sample

The research subjects were over 18 years old, with non-specific CU, of both sexes and age groups, registered as patients at the teaching clinic of this study. Those immunosuppressed due to genetic causes, psychiatric disorders and/or previous scar treatment were excluded. The sampling process was intentional. The school clinic served 55 fixed patients registered during the study period, of which 15 were excluded for this study after analysis of eligibility criteria, resulting in a sample of 40 patients for this study.

### Study outcome

Participants who met the inclusion criteria were divided into equal parts, blinded and randomly allocated into two groups: control group – conventional care plus essential fatty acid (EFA) enriched with vitamins A and E as primary coverage; and experimental group – conventional care plus GPO at 5% was provided as primary coverage. Both groups underwent secondary coverage, consisting of sterile gauze, with a 100% cotton crepe bandage immediately after application of primary coverage.

Study randomization was carried out using a list generated in Microsoft Office Excel®, with participants randomly distributed to create a control group using EFA and an experimental group using 5% GPO.

Allocations were concealed using separate, sequentially numbered, opaque envelopes on the outside containing information about the randomly selected groups. Procedures were carried out by a specialist who did not maintain contact with the main researcher. Envelopes were only opened at the time of requesting the intervention, to avoid clarity regarding participant allocation.

The reason for choosing EFA to conduct the control group was due to the fact that it is a commercialized and easily obtainable component for injury treatment, already used in the teaching clinic of this research. Therefore, understanding the anti-inflammatory potential, presence of linoleic acid (omega-6), alpha-linolenic acid (omega-3), vitamins A and E, EFA has topical therapeutic potential in CU treatment^(8)^. Furthermore, EFA is widely used in the clinic for shortening ulcer healing time, as it induces topical (observed by the local production of IGF-1, leptin, IL-6 and IFN-γ) and systemic effects, reducing serum levels of IL-6^(8)^.

Individuals were monitored and treated for a period of 30 days. Conventional assistance was provided to both groups daily, which consisted of cleaning the injuries with 0.9% saline solution, followed by sterile gauze use to remove dirt, always rubbed from the center of the injury to its periphery. Soon after, applications of EFA enriched with vitamins A and E (control group) or 5% GPO (experimental group) were carried out, followed by secondary coverage of sterile gauze with a 100% cotton crepe bandage.

To guide data collection, CU photographs were taken and consultations were made in the medical records made available by the school clinic, in order to collect information regarding sociodemographic profile (sex, age, marital status, education, profession, type of residence and whether they have access to basic sanitation) and clinical-laboratory findings (type and degree of injury, hemoglobin, leukocytes, platelets, glucose and albumin). Furthermore, injury morphological characteristics were measured (type of tissue in the bed, type of exudate, edge characteristic and regularity, presence of microbial content and pain), through using instruments previously designed based on the main macroscopic signs and symptoms associated with the healing process.

The GPO used in this study was produced and provided by the company *BaldoniProdutos Naturais.* As stated in the technical sheet, to prepare the GPO, green propolis extracts were added at a concentration of 5% of its weight, in a lanovaseline-based ointment (simple ointment) composed of 30% lanolin, 0.02% butyl hydroxytoluene (BHT) and 100% solid petroleum jelly (q.s.p.). In this study, only GPO samples that passed quality tests were used^(9)^.

The entire intervention process was carried out by a nurse specializing in dermatology, individually, in a private space provided by the school clinic, in order to guarantee participant safety and privacy. Data collection occurred through two nurses, validated by a third professional to guarantee data reliability.

Sociodemographic and clinical characteristics variables were collected with information on biological sex, age, marital status, education, profession, type of residence, basic sanitation, type of injury, degree of injury and whether a patient is diabetic. At blood levels, data on hemoglobin, leukocytes, platelets, albumin and glucose were collected. In proportion to lesion characteristics, information was collected on the type of tissue in the bed, type of exudate, edge regularity, edge characteristics, microbial content and pain.

Photographic records were taken daily, always after cleaning the injury, placing a ruler next to the wound in parallel with healthy skin, using a smartphone camera with an effective static resolution of 48 megapixels. To measure the injury area, the captured images were digitally transferred to a notebook and processed in the Image J freeware (version 1.45s, National Institutes of Health, Rockville, MD), according to the method proposed by Aragón-Sánchez^(10)^.

### Analysis of results, and statistics

The percentage of wound contraction (%WC) was calculated using the mathematical equation: %WC = (Ia-Fa)/Iax100, with Ia = initial area (injury area before application of the test substance) and Fa = final area (injury area after 30 days of test substance application)^(11)^.

Descriptive statistics (means and standard deviations) were used for continuous variables, and frequency distributions (absolute and relative values) for categorical variables. Data normality was verified by the Kolmogorov-Smirnov and Shapiro-Wilk tests, and homogeneity by Levene’s test. Bootstrapping procedures (1,000 resamples; 95% CI BCa) were implemented to obtain greater reliability of the results, to correct deviations from normality of sample distribution and differences between group sizes and, also, to present a 95% Confidence Interval for differences between means^(11)^.

To compare age between the control and experimental groups, t-test for independent samples was used. The remaining categorical variables were compared between patients who received control and experimental treatment using Pearson’s chi-square test or Fisher’s exact test.

A factorial ANOVA (2x2) was carried out with the aim of verifying to what extent the %WC (percentage of wound contraction) was different between the type of treatment (control and experimental group) and the fact of being diabetic or not. Post-hoc analyzes for main and interaction effects (diabetic*group) were performed using the Bonferroni test.

The McNemar test was applied in order to investigate whether the proportions of lesion characteristics (type of tissue in the bed, presence of exudate, edge regularity, edge characteristics, microbial content and pain) were equivalent for each treatment group (control and experimental) between the two moments assessed.

For hypothesis tests in the different statistical models, a p-value < .05 was considered significant. All analyzes were performed using the Statistical Package for the Social Sciences (SPSS) (version 25).

## RESULTS

In the control group (N=20), participants had a mean age of 71.50±18.05 years, the majority were female (65%), married (50%), illiterate (85%), with their own home (90%) and with access to basic sanitation (95%). With regard to injuries, it was observed that, in the control group, 55% of the individuals were not diabetic and the majority had pressure injuries (40%) and wounds classified as degree III (55%). In the experimental group (N=20), participants had a mean age of 71.05±18.91 years, the majority were male (60%), married (55%), illiterate (85%) and retired (75%).

As for housing, 100% of individuals had their own home and the majority had access to basic sanitation (95%). As for clinical variables, 60% of the experimental group sample was made up of diabetics, victims of pressure injuries (45%), with wounds classified as degree II (45%). When comparing the profiles of participants in the different groups (control and experimental), it was observed that there was no statistically significant difference for the variables tested (p > .05). Table 1 presents participant sociodemographic and clinical characteristics in each treatment group (control and experimental).

**Table 1:** Participant sociodemographic and clinical characteristics. Sousa, Paraíba, Brazil, 2023

Variable	M (SD) or n (%)		*t* or χ^2^ (df)
	Control (N=20)	Experimental (N=20)	
Sex			
Male	7 (35)	12 (60)	2.51 (1)^ns^
Female	13 (65)	8 (40)	
Age (years)	71.50±18.05	71.05±18.91	0.08 (38)^ns^
Marital status			
Married	10 (50)	11 (55)	1.05 (3)^ns^
Widower	6 (30)	6 (30)	
Divorced	1 (5)	0 (0)	
Single	3 (15)	3 (15)	
Education			
Illiterate	17 (85)	14 (70)	6.62 (5)^ns^
Literate	1 (5)	2 (10)	
Incomplete elementary school	2 (10)	0 (0)	
Complete elementary school	0 (0)	1 (5)	
High school	0 (0)	2 (10)	
Higher education	0 (0)	1 (5)	
Profession			
Retiree	15 (75)	12 (60)	6.00 (8)^ns^
Sickness benefit	1 (5)	2 (10)	
Farmer	1 (5)	2 (10)	
Cowboy	1 (5)	0 (0)	
Autonomous	0 (0)	1 (5)	
Attorney	0 (0)	1 (5)	
Machine operator	0 (0)	1 (5)	
From home	1 (5)	0 (0)	
Did not answer	1 (5)	1 (5)	
Type of residence			
Own	18 (90)	20 (100)	2.11 (2)^ns^
Shelter	1 (5)	0 (0)	
Settlement	1 (5)	0 (0)	
Basic sanitation			
Yes	19 (95)	19 (95)	0.00 (1)^ns^
No	1 (5)	1 (5)	
Type of injury			
Carcinoma characteristic	1 (5)	3 (15)	7.42 (8)^ns^
Pressure injury	8 (40)	8 (40)	
Diabetic foot ulcer	5 (25)	2 (10)	
Venous ulcer	1 (5)	4 (20)	
Varicose ulcer	1 (5)	1 (5)	
Mixed ulcer	1 (5)	0 (0)	
Erythematous	1 (5)	0 (0)	
Bullous erysipelas	0 (0)	1 (5)	
Surgical dehiscence	2 (10)	1 (5)	
Injury degree			
II	6 (30)	9 (45)	1.07 (2)^ns^
III	11 (55)	8 (40)	
IV	3 (15)	3 (15)	
Diabetic			
Yes	9 (45)	12 (60)	0.90 (1)^ns^
No	11 (55)	8 (40)	

*Note: t-t test; χ^2^ – chi-square; df – degrees of freedom; ns – not significant; SD – standard deviation.*

Concerning laboratory tests, t-tests for independent samples demonstrated that there was no statistically significant difference when comparing blood levels of hemoglobin, leukocytes, platelets, albumin and glucose between the different treatment groups (Table 2).

**Table 2:** Comparisons of blood levels of hemoglobin, leukocytes, platelets, albumin and glucose between different treatment groups (control and experimental). Sousa, Paraíba, Brazil, 2023

Variables	Group	M (SD)	Difference of mean	Confidence Interval (95% BCa)		*t* (df)
				Lower	Upper	
Hemoglobin	Control	11.55 (1.43)	−.81	10.86	12.15	−1.95 (38)^ns^
	Experimental	12.36 (1.19)		11.88	12.84	
Leukocytes	Control	7.1 (2.96)	−.55	6.15	8.15	−.72 (38)^ns^
	Experimental	7.64 (1.64)		6.95	8.38	
Platelets	Control	246.75 (57.24)	−4.35	217.12	275.47	−.22 (38)^ns^
	Experimental	251.1 (57.25)		229.74	278.25	
Glucose	Control	104.2 (35.83)	−5.48	91.16	120.23	−.4 (38)^ns^
	Experimental	109.68 (50.03)		90.75	133.15	
Albumin	Control	3.77 (.73)	.299	3.45	4.07	1.21 (38)^ns^
	Experimental	3.47 (.83)		3.09	3.77	

*Note: BCa – bias corrected accelerated; t–t test; df – degrees of freedom; M – mean; SD – standard deviation; ns – not significant.*

Table 3 presents descriptive statistics of wound contraction percentages for all groups.

**Table 3:** Descriptive statistics of the percentage of wound contraction (%WC) for the treatment groups (control and experimental). Sousa, Paraíba, Brazil, 2023

Group	Diabetic	M (SD)	Confidence Interval (95% BCa)	
			Lower	Upper
Control	Yes	47.27 (20.48)	34.35	60.96
	No	24.98 (51.53)	−14.96	56.56
	Total	35.01 (41.28)	12.69	54.05
Experimental	Yes	19.05 (49.69)	−21.64	43.58
	No	32.63 (25.79)	16.16	52.43
	Total	24.49 (41.49)	1.69	40.88
Total	Yes	31.15 (41.61)	8.76	47.77
	No	28.21 (41.82)	4.35	47.42
	Total	29.75 (41.19)	15.09	43.01

*Note: BCa − bias corrected accelerated; M – mean; SD – standard deviation.*

The factorial ANOVA (2×2) results demonstrated that there was no statistically significant effect for the type of treatment (*F* (1, 36) = .601, *p* >.05, χ^2^ = 0.016) nor for the fact of being diabetic or not (*F* (1, 36) = .108, *p* >.05, η^2^ = 0.003) as well as for the interaction between these two variables (*F* (1, 36) = 1.829, *p* > .05, η^2^ = 0.048). Multiple comparison analyzes (Bonferroni post-hoc) are presented in Table 4.

**Table 4:** Multiple comparisons (Bonferroni post-hoc with bootstrapping procedures) of the percentage of wound contraction (%WC) for the main effects (type of treatment and being or not being diabetic) and for interaction effects (treatment*diabetic). Sousa, Paraíba, Brazil, 2023

	Compared groups		Difference of mean	Confidence Interval (95% BCa)	
				Lower	Upper
Main effects					
Treatment	Control	Experimental	10.282^ns^	−11.27	33.99
Diabetic	Yes	No	4.35^ns^	−21.69	26.09
Effects of interaction (Diabetic*treatment)					
Treatment	Diabetic				
Control	Yes	No	22.29^ns^	−15.46	60.04
Experimental	Yes	No	−13.58^ns^	−51.92	24.75
Diabetic	Treatment				
Yes	Control	Experimental	28.2^ns^	−8.82	65.25
No	Control	Experimental	-7.66^ns^	−31.37	46.68

*Note: BCa – bias corrected accelerated; ns – not significant.*

Regarding the effect of treatment on lesion characteristics, the McNemar test found significant differences for the group treated with the control in the proportions of type of exudate (χ^2^(1) = 5.14, p < .05) and microbial content (χ^2^(1) = 7.11, p < .001), indicating that, on day 30, there was a greater presence of serous exudate in injuries and a smaller number of infected wounds. For the experimental group, significant results were found for the type of tissue in the bed (χ^2^(1) = 9.09, p < .001), type of exudate (χ^2^(1) = 7.11, p < .001), edge characteristics (χ^2^(1) = 4.00, p < .01), microbial content (χ^2^(1) = 11.08, p < .0001) and pain (χ^2^(1) = 5.14, p < .01), demonstrating that, when comparing lesion characteristics between the two moments assessed, on the last day of assessment, there were injuries with greater proportions of healing tissue, greater presence of serous exudate and with more intact edges, absence of infected wounds and a lower pain intensity.

For the control group, a difference in the proportions of type of exudate and microbial content was identified. For the experimental group, in the type of tissue in the bed, type of exudate, edge characteristics, microbial content and pain, it was demonstrated that, when comparing lesion characteristics between the two moments assessed, on the last day of assessment, there were injuries with greater proportions of healing tissue, greater presence of serous exudate and with more intact edges, absence of infected wounds and a lower pain intensity (Table 5).

**Table 5:** Comparisons of proportions of lesion characteristics on day 0 and day 30 (type of tissue in the bed, presence of exudate, edge regularity, edge characteristics, microbial content and pain) for each treatment group (control and experimental). Sousa, Paraíba, Brazil, 2023

Lesion characteristics	Control group n (%)		*X* ^2^ (df)	Experimental group n (%)		*X* ^2^ (df)
	Day 0	Day 30		Day 0	Day 30	
Type of tissue in the bed			2.29 (1)^ns^			9.09 (1)**
Healing	10 (50)	15 (75)		7 (35)	18 (90)	
Necrotic	10 (50)	5 (25)		13 (65)	2 (10)	
Type of exudate			5.14 (1)*			7.11 (1)**
Serous	9 (45)	16 (80)		10 (50)	19 (95)	
Purulent	11 (55)	4 (20)		10 (50)	1 (5)	
Edge regularity			.80 (1)^ns^			2.25 (1)^ns^
Regular	9 (45)	12 (60)		7 (35)	3 (85)	
Irregular	11 (55)	8 (40)		13 (65)	17 (85)	
Edge characteristics			.00 (1)^ns^			4.00 (1)^*^
Full	10 (50)	11 (55)		11 (55)	18 (90)	
Not full	10 (50)	9 (45)		9 (45)	2 (10)	
Microbial content			7.11 (1)^**^			11.08 (1)***
Contaminated	9 (45)	18 (90)		7 (35)	20 (100)	
Infected	11 (55)	2 (10)		13 (65)	0 (0)	
Pain			2.29 (1)^ns^			5.14 (1)*
Mild	7 (35)	12 (60)		8 (40)	15 (75)	
Moderate	13 (65)	8 (40)		12 (60)	5 (25)	

Note: ns – not significant; *p < .01; **p < .001; ***p < .0001; χ^2^=chi-square; df – degrees of freedom.

## DISCUSSION

The comparison of individuals in the control group with the experimental group showed that, in general, the two groups were similar in sociodemographic, clinical characteristics and types of injury, considering the homogeneity between the groups. Comparisons of injury proportions and variables showed evolution regarding the type of tissue in the bed, presence of exudate, edge regularity, edge characteristics, microbial content and pain in both treated groups; however, those with GPO use showed significant effects on the group control.

Injury healing rates in both groups began to converge quickly after completing treatment; however, with propolis (after week 4), a progressive difference was noticed in the type of tissue found in the bed of the treated wounds and in the reduction of infections in injuries. Propolis’ antibacterial activity is known through polyphenols and flavonoids in combating *Escherichia coli* and *Staphylococcus aureus* ^(12)^. Gram-positive bacteria action is more effective when compared to combating Gram-negative bacteria. Propolis use has no side effects, which favors its adherence^(13)^. However, recent studies on propolis’ antibacterial mechanism are not clear, but they describe greater effectiveness when associated with other biological materials in CU treatment, such as oral antibiotic use^(14)^.

There was a change from non-viable to viable tissue, i.e., the necrotic tissue gave way to granulation tissue, with subsequent neo-formation of incipient epithelial tissue, demonstrating that GPO’s cleaning capacity was effective. This is explained by its presence of enzymes that favor the healing process, with the enzyme myeloperoxidase being one of the most notable. This helps break down and dissolve unviable tissues, facilitating their removal and promoting a more conducive environment for developing granulation tissues^(15)^.

In the experimental group, continuous monitoring of scar tissue during treatment was observed, including those with a higher degree of injury and conditions that make microcirculation and local nutrition impossible. Nutrients carried by the blood make a total difference in the development of viable tissue. Individuals with circulatory difficulties, diabetes mellitus and other conditions may be harmed by a lack of nutrients for healing. Therefore, the amino acids, vitamins and carbohydrates present in propolis favor local nutrition for wound tissues, improving the tissue microenvironment when applied topically^(14)^.

It is worth noting that there was the presence of diabetic and non-diabetic individuals in this research in both groups. Multiple comparison analysis showed that there is no statistically significant effect for the type of treatment between the groups, nor for the fact of being diabetic or not, as well as for the interaction between these two variables. Therefore, the fact of containing diabetic individuals in the sample, despite the condition delaying wound healing, did not show any difference between participants and the potential effect of treatment, indicating that GPO is a healing element for both types of wounds.

CU pain reduction was one of the benefits observed for the experimental group, which may be directly associated with the power of debridement and wound cleaning with GPO use. CU is related to inflammation, which is mediated by chemical substances such as cytokines, prostaglandins and growth factors. These substances irritate the nerves near the wound, triggering pain perception. Furthermore, the presence of necrotic tissue and exudate in wounds can create an acidic environment prone to infection, worsening pain. Removing non-viable tissue and controlling inflammation are crucial approaches to reducing pain^(16)^.

Furthermore, studies show that propolis’ anti-inflammatory effect is similar to non-steroidal anti-inflammatory drugs, presenting beneficial action on fibronectin metabolism by inhibiting the biosynthesis of native fibronectin to reduce its degradation in damaged tissue^(17)^. It has also been shown that in burn wounds treated with GPO, free radical concentration is lower than in wounds treated with silver sulfadiazine^(18)^.

### Contributions to nursing, health or public policy

As a contribution to the advancement in nursing care practice, this study presents important results of a natural resource that expresses effectiveness and can be used in nursing care for CU treatment. With the advancement of technological resources, numerous products are developed and tested for CU treatment; however, it is known that cost-benefit is also a factor that must be assessed when choosing the product. Therefore, Brazilian green propolis is a reliable and proven option in terms of its effectiveness, as it is easy to access and low cost, and can be implemented directly in care.

Furthermore, this study paves the way for the development of new research, using new comparison techniques and presenting evidence in a systematic way, as nursing is a science that seeks to implement evidence-based practice in its care.

### Study limitations

Sample size, research duration, patient exclusion (immuno-suppressed due to genetic causes, psychiatric disorders and/or undergoing previous scar treatment) and the fact that the study is unicentric can be considered as limitations of this study.

## CONCLUSIONS

This research was able to present data about the effects of Brazilian green propolis on CU treatment. Their results show that this resource has healing potential and allows the degradation of non-viable tissue, leading to the appearance of granulation tissue. Changes in edge characteristics, reduction in pain and reduction in microbial load were also identified, enabling a progressive difference in the type of tissue found in the treated CU bed as well as in patients with associated conditions, such as diabetes *mellitus.*

When compared with the control population, it is suggested that propolis possibly has a better healing effect than EFA, presenting more efficacy in preparing the CU bed in clinical and macroscopic aspects, suggesting it to be a CU cleaning and healing product with a debriding effect.
