# Supplementary material for: Effect of Brazilian green propolis in chronic ulcer treatment: a randomized clinical trial
Source: Rev Bras Enferm. 2024 Sep 6;77(4):e20230418. doi: 10.1590/0034-7167-2023-0418 (PMC11382678; doi:10.1590/0034-7167-2023-0418)
Supplement: Translation

# Translation: Efeito da própolis verde brasileira no tratamento de úlceras crônicas: ensaio clínico randomizado

**DOI:** 10.1590/0034-7167-2023-0418pt

**Authors:** Pascalle de Sousa Rocha, Luis Rafael Leite Sampaio, Gyllyandeson de Araújo Delmondes, Andreza Gysllaynny Delmondes Saraiva, Fábio Ferreira Perazzo, Fernando Luiz Affonso Fonseca, José Lucas de Souza, Luis Fernando Reis Macedo

**Affiliations:** I Faculdade de Medicina do ABC. Santo André, São Paulo, Brasil; II Universidade Federal de São Paulo. São Paulo, São Paulo, Brasil; III Instituto Federal da Paraíba. Sousa, Paraíba, Brasil; IV Universidade Regional do Cariri. Crato, Ceará, Brasil; V Universidade Federal do Vale do São Francisco. Petrolina, Pernambuco, Brasil; VI Faculdade de Medicina Estácio de Juazeiro do Norte. Juazeiro do Norte, Ceará, Brasil

**Keywords:** Úlcera, Ferimentos e Lesões, Produtos Biológicos, Ensaio Clínico, Estomaterapia

## Abstract

**Objetivo:**

avaliar a eficácia da própolis verde brasileira a 5% (pomada) em indivíduos com úlceras crônicas.

**Métodos:**

ensaio clínico randomizado, desenvolvido com 40 pacientes aleatorizados igualmente para grupo controle (tratado com ácido graxo essencial) e grupo experimental (tratado com própolis verde a 5%) por 30 dias. Os desfechos de interesse foram características sociodemográficas, clínicas e laboratoriais, características das lesões, como tipo de tecido no leito, presença de exsudato, características da borda, conteúdo microbiano e dor.

**Resultados:**

quanto às características sociodemográficas, clínicas e laboratoriais, os dois grupos não apresentaram diferença estatisticamente significativas. Após avaliação em 30 dias, observou-se efeito para os dois grupos tratados, porém, para o grupo experimental, maior eficácia quanto ao tipo de tecido no leito, tipo de exsudato, característica da borda, conteúdo microbiano e dor.

**Conclusão:**

a pomada a base de própolis mostrou efeito cicatrizante, apresentando-se como potencial instrumento na cicatrização de úlceras crônicas.

## INTRODUÇÃO

As úlceras crônicas (UC) são um crescente problema de saúde pública que afeta milhões de pessoas em todo o mundo. Podem variar em gravidade e causa, frequentemente resultantes de problemas subjacentes, como distúrbios circulatórios/metabólicos ou condições autoimunes, o que torna o tratamento muito mais desafiador, impactando significativamente a qualidade de vida dos afetados^(1)^.

Condições associadas a essa causa levam ao prolongamento da cicatrização e ao aumento de custos para o serviço de saúde e para o paciente. Estima-se que, para os últimos anos, haja gradação da incidência e prevalência de indivíduos com UC associada a condições crônicas-degenerativas. Portanto, estudos recentes apontam que a prevalência de UC de etiologia mista é de 2,21 por 1.000 indivíduos^(2)^.

Com isso, estratégias são desenvolvidas continuamente, como tecnologias, novas condutas terapêuticas, extração de matérias e recursos naturais, entre outros, para o tratamento das UC, possibilitando uma rápida ação para as necessidades do paciente e evitando complicações^(3)^. Por isso, a Política Nacional de Práticas Integrativas e Complementares atua na utilização de recursos terapêuticos, não derivados de medicamentos, que busca reconhecer a tradição do cuidado humano, validando e incluindo o uso de produtos biológicos como forma de intervenção para uma série de condições em saúde^(4)^.

Desse modo, a própolis verde brasileira é um recurso natural conhecido por suas propriedades químicas e farmacológicas que podem ser benéficas no tratamento de UC. Trata-se de resina produzida por abelhas a partir de substâncias vegetais. No entanto, é importante observar que a composição da própolis pode variar dependendo da região geográfica e das plantas locais das quais as abelhas a obtêm^(5)^.

A própolis brasileira é rica em compostos bioativos que exibem diversas propriedades terapêuticas, tais como propriedades antimicrobianas, anti-inflamatórias, antioxidantes, analgésicas, entre outras. Ela tem sido associada à aceleração do processo de cicatrização de UC, estimulando também a produção de colágeno, além de promover a angiogênese^(6)^.

Dessa forma, percebe-se a importância de explorar os benefícios do uso da própolis brasileira para o tratamento de UC. Assim, deu-se seguimento à seguinte pergunta-problema: indivíduos com UC tratados com pomada de própolis verde (PPV) a 5% terão maior percentual de cicatrização do que aqueles tratados com terapia convencional?

## OBJETIVO

Avaliar a eficácia do uso da PPV a 5% no tratamento de indivíduos com UC.

## MÉTODOS

### Aspectos éticos

O processo de intervenção e a coleta de dados ocorreram mediante aprovação em Comitê de Ética em Pesquisa (CEP) e o cadastro no Registro Brasileiro de Ensaios Clínicos (ReBEC) (nº RBR-294d68). Todas as fases da pesquisa seguiram rigorosamente as exigências éticas e legais da Resolução nº 466/2012 do Conselho Nacional de Saúde/Ministério da Saúde. Ressalta-se que este estudo seguiu os protocolos necessários para atender ao participante e a seus familiares, respeitando-os quanto à autonomia e defendendo-os em sua vulnerabilidade, através da assinatura do Termo de Consentimento Livre Esclarecido, garantido o sigilo das informações e a liberdade de participação no grupo.

### Desenho, local e período do estudo

Trata-se de ensaio clínico randomizado controlado e unicego, desenvolvido em conformidade com as diretrizes do *CONsolidated Standards Of Reporting Trials* (CONSORT)^(7)^, realizado em uma Clínica Escola de Enfermagem na cidade de Cajazeiras (06º53’24 “S; 38°33’43”W), Paraíba, Brasil. O estudo foi realizado de março a outubro de 2019.

### População e amostra

Os sujeitos da pesquisa foram maiores de 18 anos, com UC inespecíficas, de ambos os sexos e faixas etárias, cadastrados como pacientes na clínica escola do presente estudo. Foram excluídos aqueles imunossuprimidos por causas genéticas, transtornos psiquiátricos e/ou em tratamento cicatricial prévio. O processo de amostragem foi do tipo intencional. A clínica escola atendeu 55 pacientes fixos registrados no período do estudo, no qual 15 foram excluídos para este estudo após análise de critérios de elegibilidade, tendo uma amostragem de 40 pacientes para este estudo.

### Desfecho do estudo

Os participantes que atenderam aos critérios de inclusão foram divididos em partes iguais, cegados e alocados aletoriamente em dois grupos: grupo controle – realizada assistência convencional mais ácido graxo essencial (AGE) enriquecido com vitaminas A e E como cobertura primária; e grupo experimental – realizada assistência convencional mais PPV a 5% como cobertura primária. Ambos os grupos foram submetidos à cobertura secundária, composta por gaze estéril, com faixa do tipo atadura de crepom 100% algodão imediatamente após a aplicação da cobertura primária.

A randomização do estudo foi realizada por meio de lista gerada no programa Microsoft Office Excel®, sendo os participantes distribuídos aleatoriamente para criar um grupo controle utilizando AGE e um grupo experimental utilizando PPV a 5%.

As alocações foram ocultadas por meio de envelopes opacos separados, numerados sequencialmente, na parte externa, contendo informações sobre os grupos selecionados aleatoriamente. Os procedimentos foram realizados por especialista que não manteve contato com o pesquisador principal. Os envelopes foram abertos apenas no momento da solicitação da intervenção, para evitar clareza sobre a alocação dos participantes.

O motivo da escolha do AGE na condução do grupo controle deu-se pelo fato de ser um componente comercializado e de fácil obtenção para o tratamento de lesões, já utilizado na clínica escola desta pesquisa. Portanto, compreendendo o potencial anti-inflamatório, presença de ácido linoleico (ômega-6), ácido alfa-linolênico (ômega-3), vitaminas A e E, tem-se o AGE como potencial terapêutico tópico no tratamento de UC^(8)^. Ademais, o AGE é amplamente utilizado na clínica por encurtar o tempo de cicatrização de úlceras, visto que induz efeitos tópicos (observados pela produção local de IGF-1, leptina, IL-6 e IFN-γ) e sistêmicos, reduzindo os níveis séricos de IL-6^(8)^.

Os indivíduos foram acompanhados e tratados por um período de 30 dias. A assistência convencional foi realizada aos dois grupos diariamente, que constava da limpeza das lesões com solução fisiológica a 0,9%, seguida do uso de gaze estéril para retirada de sujidades, sempre friccionada do centro da lesão até a sua periferia. Logo após, foram realizadas as aplicações do AGE enriquecido com vitaminas A e E (grupo controle) ou PPV a 5% (grupo experimental), seguidas de cobertura secundária de gaze estéril com faixa do tipo atadura de crepom 100% algodão.

Para guiar a coleta de dados, foram realizadas fotografias das UC, consultas nos prontuários disponibilizados pela clínica escola, a fim de coletar informações referentes ao perfil sociode-mográfico (sexo, idade, estado civil, escolaridade, profissão, tipo de residência e se tem acesso a saneamento básico) e achados clínico-laboratoriais (tipo e grau da lesão, hemoglobina, leucócitos, plaquetas, glicose e albumina). Além disso, foram mensuradas as características morfológicas das lesões (tipo de tecido no leito, tipo de exsudato, característica e regularidade da borda, presença de conteúdo microbiano e dor), mediante o uso de instrumentos previamente elaborados com base nos principais sinais e sintomas macroscópicos associados ao processo de cicatrização.

A PPV utilizada neste estudo foi produzida e cedida pela empresa Baldoni Produtos Naturais. Conforme consta na ficha técnica, para preparação da PPV, foram adicionados extratos de própolis verde em uma concentração de 5% do seu peso, em uma pomada a base de lanovaselina (pomada simples) composta por lanolina a 30%, hidroxitolueno de butila (BHT) a 0,02% e vaselina sólida a 100% (q.s.p.). Neste estudo, foram utilizadas somente as amostras da PPV que passaram nos testes de qualidade^(9)^.

Todo o processo de intervenção foi realizado por um enfermeiro especialista em dermatologia, de forma individual, em local reservado cedido pela clínica escola, a fim de garantir a segurança e a privacidade dos participantes. A coleta de dados ocorreu através de dois enfermeiros, validada por um terceiro profissional como garantia da fidedignidade dos dados.

Foram coletadas variáveis de características sociodemográficas e clinicas com informações sobre sexo biológico, idade, estado civil, escolaridade, profissão, tipo de residência, saneamento básico, tipo de lesão, grau da lesão e se é diabético. Em níveis sanguíneos, foram coletados dados sobre hemoglobina, leucócitos, plaquetas, albumina e glicose. Em proporção das características da lesão, foram coletadas informações sobre tipo de tecido no leito, tipo de exsudato, regularidade da borda, característica da borda, conteúdo microbiano e dor.

Os registros fotográficos foram realizados diariamente, sempre após a limpeza da lesão, colocando uma régua ao lado da ferida em paralelo com a pele saudável, utilizando uma câmera fotográfica de *smartphone* com resolução estática efetiva de 48 *megapixels.* Para mensuração da área da lesão, as imagens captadas eram transferidas digitalmente para um *notebook* e processadas no *freeware Image J* (versão 1.45s, *National Institutes of Health,* Rockville, MD), conforme o método proposto por Aragón-Sánchez^(10)^.

### Análise dos resultados e estatística

O percentual de contração da ferida (%CF) foi calculado através da equação matemática: %CF = (Ai-Af)/Aix100, sendo Ai = área inicial (área da lesão antes da aplicação da substância teste) e Af = área final (área da lesão após 30 dias de aplicação da substância teste)^(11)^.

Estatísticas descritivas (médias e desvios padrão) foram utilizadas para as variáveis contínuas, e distribuições de frequência (valores absolutos e relativos), para as variáveis categóricas. A normalidade dos dados foi verificada pelos testes Kolmogoro-v-Smirnov e Shapiro-Wilk, e a homogeneidade, pelo teste de Levene. Procedimentos de *bootstrapping* (1.000 reamostragens; 95% IC BCa) foram implementados para se obter uma maior confiabilidade dos resultados, para corrigir desvios de normalidade da distribuição da amostra e diferenças entre os tamanhos dos grupos e, também, para apresentar um Intervalo de Confiança de 95% para as diferenças entre as médias^(11)^.

Para comparar a idade entre o grupo controle e experimental, foi utilizado o teste *t* para amostras independentes. As demais variáveis categóricas foram comparadas entre pacientes que receberam o tratamento controle e experimental por meio do teste do qui-quadrado de Pearson ou teste exato de Fisher.

Foi realizada uma ANOVA fatorial (2×2) com o objetivo de verificar em que medida o %CF (percentual de contração da ferida) era diferente entre o tipo de tratamento (grupo controle e experimental) e o fato ser ou não ser diabético. As análises de *post-hoc* para os efeitos principais e de interação (grupo*-diabético) foram realizados por meio do teste de Bonferroni.

O teste de McNemar foi aplicado a fim de investigar se as proporções das características da lesão (tipo de tecido no leito, presença de exsudato, regularidade da borda, característica da borda, conteúdo microbiano e dor) eram equivalentes, para cada grupo de tratamento (controle e experimental), entre os dois momentos avaliados.

Para os testes de hipóteses nos diferentes modelos estatísticos, valor de p < ,05 foi considerado significativo. Todas as análises foram realizadas no *software Statistical Package for the Social Sciences* (SPSS) (versão 25).

## RESULTADOS

No grupo controle (N=20), os participantes tinham idade média de 71,50±18,05 anos, sendo a maioria do sexo feminino (65%), casada (50%), analfabeta (85%), com moradia própria (90%) e com acesso a saneamento básico (95%). No que se refere às lesões, observou-se que, no grupo controle, 55% dos indivíduos não eram diabéticos e a maioria apresentava lesões por pressão (40%) e feridas classificadas como grau III (55%). Já no grupo experimental (N=20), os participantes tinham idade média de 71,05±18,91 anos, sendo a maioria do sexo masculino (60%), casada (55%), analfabeta (85%) e aposentada (75%).

Quanto à moradia, 100% dos indivíduos tinham casa própria e a maioria tinha acesso a saneamento básico (95%). Quanto às variáveis clínicas, 60% da amostra do grupo experimental era composta por diabéticos, vítimas de lesão por pressão (45%), com feridas classificadas como grau II (45%). Ao se comparar os perfis dos participantes dos diferentes grupos (controle e experimental), observou-se que não houve diferença estatisticamente significativa para as variáveis testadas (p > ,05). A Tabela 1 apresenta as características sociodemográficas e clínicas dos participantes de cada grupo de tratamento (controle e experimental).

**Tabela 1:** Características sociodemográficas e clínicas dos participantes. Sousa, Paraíba, Brasil, 2023

Variável	M (DP) ou n (%)		*t* ou χ^2^ (gl)
	Controle (N=20)	Experimental (N=20)	
Sexo			
Masculino	7 (35)	12 (60)	2,51 (1)^ns^
Feminino	13 (65)	8 (40)	
Idade (anos)	71,50±18,05	71,05±18,91	0,08 (38)^ns^
Estado civil			
Casado(a)	10 (50)	11 (55)	1,05 (3)^ns^
Viúvo(a)	6 (30)	6 (30)	
Divorciado(a)	1 (5)	0 (0)	
Solteiro(a)	3 (15)	3 (15)	
Escolaridade			
Analfabeto(a)	17 (85)	14 (70)	6,62 (5)^ns^
Alfabetizado(a)	1 (5)	2 (10)	
Fundamental incompleto	2 (10)	0 (0)	
Fundamental completo	0 (0)	1 (5)	
Ensino médio	0 (0)	2 (10)	
Ensino superior	0 (0)	1 (5)	
Profissão			
Aposentado	15 (75)	12 (60)	6,00 (8)^ns^
Auxílio doença	1 (5)	2 (10)	
Agricultor	1 (5)	2 (10)	
Vaqueiro	1 (5)	0 (0)	
Autônomo	0 (0)	1 (5)	
Advogado	0 (0)	1 (5)	
Operador de máquina	0 (0)	1 (5)	
Do lar	1 (5)	0 (0)	
Não respondeu	1 (5)	1 (5)	
Tipo de residência			
Própria	18 (90)	20 (100)	2,11 (2)^ns^
Abrigo	1 (5)	0 (0)	
Assentamento	1 (5)	0 (0)	
Saneamento básico			
Sim	19 (95)	19 (95)	
Não	1 (5)	1 (5)	0,00 (1)^ns^
Tipo de lesão			
Característica de carcinoma	1 (5)	3 (15)	7,42 (8)^ns^
Lesão por pressão	8 (40)	8 (40)	
Úlcera em pé diabético	5 (25)	2 (10)	
Úlcera venosa	1 (5)	4 (20)	
Úlcera varicosa	1 (5)	1 (5)	
Úlcera mista	1 (5)	0 (0)	
Eritematosa	1 (5)	0 (0)	
Erisipela bolhosa	0 (0)	1 (5)	
Deiscência cirúrgica	2 (10)	1 (5)	
Grau da lesão			
II	6 (30)	9 (45)	1,07 (2)^ns^
III	11 (55)	8 (40)	
IV	3 (15)	3 (15)	
Diabéticos			
Sim	9 (45)	12 (60)	0,90 (1)^ns^
Não	11 (55)	8 (40)	

*Nota: t – teste t;χ^2^ – qui-quadrado;gl – graus de liberdade;ns – não significativo; DP – desvio padrão.*

No que se refere aos exames laboratoriais, os testes *t* para amostras independentes demonstraram que não houve diferença estatisticamente significativa ao se comparar os níveis sanguíneos de hemoglobina, leucócitos, plaquetas, albumina e glicose entre os diferentes grupos de tratamento (Tabela 2).

**Tabela 2:** Comparações dos níveis sanguíneos de hemoglobina, leucócitos, plaquetas, albumina e glicose entre os diferentes grupos de tratamento (controle e experimental). Sousa, Paraíba, Brasil, 2023

Variáveis	Grupo	MD (DP)	Diferença de média	Intervalo de Confiança (95% BCa)		*t*(gl)
				Inferior	Superior	
Hemoglobina	Controle	11,55 (1,43)	−,81	10,86	12,15	−1,95 (38)^ns^
	Experimental	12,36 (1,19)		11,88	12,84	
Leucócitos	Controle	7,1 (2,96)	−,55	6,15	8,15	−,72 (38)^ns^
	Experimental	7,64 (1,64)		6,95	8,38	
Plaquetas	Controle	246,75 (57,24)	−4,35	217,12	275,47	−,22 (38)^ns^
	Experimental	251,1 (57,25)		229,74	278,25	
Glicose	Controle	104,2 (35,83)	−5,48	91,16	120,23	−,4 (38)^ns^
	Experimental	109,68 (50,03)		90,75	133,15	
Albumina	Controle	3,77 (,73)	,299	3,45	4,07	1,21 (38)^ns^
	Experimental	3,47 (,83)		3,09	3,77	

*Nota: BCa – bias corrected accelerated; t – teste t; gl – graus de liberdade; MD – média; DP – desvio padrão; ns – não significativo.*

A Tabela 3 apresenta as estatísticas descritivas dos percentuais de contração da ferida para todos os grupos.

**Tabela 3:** Estatísticas descritivas do percentual de contração da ferida (%CF) para os grupos de tratamentos (controle e experimental). Sousa, Paraíba, Brasil, 2023

Grupo	Diabético	MD (DP)	Intervalo de Confiança (95% BCa)	
			Inferior	Superior
Controle	Sim	47,27 (20,48)	34,35	60,96
	Não	24,98 (51,53)	−14,96	56,56
	Total	35,01 (41,28)	12,69	54,05
Experimental	Sim	19,05 (49,69)	−21,64	43,58
	Não	32,63 (25,79)	16,16	52,43
	Total	24,49 (41,49)	1,69	40,88
Total	Sim	31,15 (41,61)	8,76	47,77
	Não	28,21 (41,82)	4,35	47,42
	Total	29,75 (41,19)	15,09	43,01

*Nota: BCa – bias corrected accelerated;MD – média; DP – desvio padrão.*

Os resultados da ANOVA fatorial (2×2) demonstraram que não houve efeito estatisticamente significativo para o tipo de tratamento *(F*(1, 36) = ,601, *p* >,05,χ^2^ = 0,016), nem para o fato de ser ou não diabético (*F* (1, 36) = ,108, *p* >,05, η^2^ = 0,003), bem como para a interação entre essas duas variáveis (*F* (1, 36) = 1,829, *p* > ,05, η^2^ = 0,048). As análises de comparações múltiplas (*post-hoc* de Bonferroni) são apresentadas na Tabela 4.

**Tabela 4:** Comparações múltiplas (*post-hoc* de *Bonferroni* com procedimentos de *bootstrapping*) do percentual de contração da ferida (%CF) para os efeitos principais (tipo de tratamento e ser ou não ser diabético) e para os efeitos de interação (tratamento*diabético). Sousa, Paraíba, Brasil, 2023

	Grupos comparados		Diferença de média	Intervalo de Confiança (95% BCa)	
				Inferior	Superior
Efeitos principais					
Tratamento	Controle	Experimental	10,282^ns^	−11,27	33,99
Diabético	Sim	Não	4,35^ns^	−21,69	26,09
Efeitos de interação (Tratamento*diabético)					
Tratamento	Diabético				
Controle	Sim	Não	22,29^ns^	−15,46	60,04
Experimental	Sim	Não	−13,58^ns^	−51,92	24,75
Diabético	Tratamento				
Sim	Controle	Experimental	28,2^ns^	−8,82	65,25
Não	Controle	Experimental	−7,66^ns^	−31,37	46,68

*Nota: BCa – bias corrected accelerated; ns – não significativo.*

No que se refere ao efeito do tratamento sobre as características da lesão, o teste de McNemar encontrou diferenças significativas para o grupo tratado com o controle nas proporções do tipo de exsudato (χ^2^(1) = 5,14, p < ,05) e conteúdo microbiano (x^2^(1) = 7,11, p < ,001), indicando que, no dia 30, havia uma maior presença de exsudato seroso nas lesões e uma menor quantidade de feridas infectadas. Para o grupo experimental, foram encontrados resultados significativos para o tipo de tecido no leito (χ^2^(1) = 9,09, p < ,001), tipo de exsudato (χ^2^(1) = 7,11, p < ,001), característica da borda (χ^2^(1) = 4,00, p < ,01), conteúdo microbiano (χ^2^(1) = 11,08, p < ,0001) e dor (χ^2^(1) = 5,14, p < ,01), demonstrando que, ao se comparar as características da lesão entre os dois momentos avaliados, no último dia de avaliação, havia lesões com maiores proporções de tecidos de cicatrização, maior presença de exsudato seroso e com bordas mais íntegras, ausência de feridas infectadas e uma menor intensidade de dor.

Identificou-se, para o grupo controle, diferença nas proporções do tipo de exsudato e conteúdo microbiano. Para o grupo experimental, no tipo de tecido no leito, tipo de exsudato, característica da borda, conteúdo microbiano e dor, demonstrou-se que, ao se comparar as características da lesão entre os dois momentos avaliados, no último dia de avaliação, havia lesões com maiores proporções de tecidos de cicatrização, maior presença de exsudato seroso e com bordas mais íntegras, ausência de feridas infectadas e uma menor intensidade de dor (Tabela 5).

**Tabela 5:** Comparações das proporções das características da lesão no dia 0 e dia 30 (tipo de tecido no leito, presença de exsudato, regularidade da borda, característica da borda, conteúdo microbiano e dor) para cada grupo de tratamento (controle e experimental). Sousa, Paraíba, Brasil, 2023

Característica da lesão	Grupo controle n (%)		χ^2^ (gl)	Grupo experimental n (%)		χ^2^(gl)
	Dia 0	Dia 30		Dia 0	Dia 30	
Tipo de tecido no leito			2,29 (1)^ns^			9,09 (1)**
Cicatrização	10 (50)	15 (75)		7 (35)	18 (90)	
Necrótico	10 (50)	5 (25)		13 (65)	2 (10)	
Tipo de exsudato			5,14 (1)*			7,11 (1)**
Seroso	9 (45)	16 (80)		10 (50)	19 (95)	
Purulento	11 (55)	4 (20)		10 (50)	1 (5)	
Regularidade da borda			,80 (1)^ns^			2,25 (1)^ns^
Regular	9 (45)	12 (60)		7 (35)	3 (85)	
Irregular	11 (55)	8 (40)		13 (65)	17 (85)	
Característica da borda			,00 (1)^ns^			4,00 (1)*
Íntegra	10 (50)	11 (55)		11 (55)	18 (90)	
Não íntegra	10 (50)	9 (45)		9 (45)	2 (10)	
Conteúdo microbiano			7,11 (1)**			11,08 (1)***
Contaminada	9 (45)	18 (90)		7 (35)	20 (100)	
Infectada	11 (55)	2 (10)		13 (65)	0 (0)	
Dor			2,29 (1)^ns^			5,14 (1)*
Leve	7 (35)	12 (60)		8 (40)	15 (75)	
Moderada	13 (65)	8 (40)		12 (60)	5 (25)	

Nota: ns – não significativo; *p < ,01; **p < ,001; ***p < ,0001; χ^2^=qui-quadrado; gl – graus de liberdade.

## DISCUSSÃO

A comparação dos indivíduos do grupo controle com o experimental mostrou que, em geral, os dois grupos eram semelhantes nas características sociodemográficas, clínicas e tipos de lesão, considerando a homogeneidade entre os grupos. As comparações das proporções e variáveis das lesões mostraram evolução quanto ao tipo de tecido no leito, presença de exsudato, regularidade da borda, característica da borda, conteúdo microbiano e dor em ambos os grupos tratados, porém aqueles com o uso da PPV apresentaram efeitos significativos ao grupo controle.

As taxas de cicatrização das lesões em ambos os grupos começaram a convergir rapidamente após a conclusão do tratamento, porém, com própolis (após a semana 4), percebeu-se um diferencial progressivo no tipo de tecido encontrado no leito das feridas tratadas e na diminuição de infecções nas lesões. A atividade antibacteriana da própolis é conhecida através dos polifenóis e flavonoides no combate do *Escherichia coli* e *Staphylococcus au*reus^(12)^. A ação em bactérias Gram-positivas é mais efetiva quando comparadas ao combate das Gram-negativas. O uso da própolis não apresenta efeitos colaterais, o que favorece a sua adesão^(13)^. Entretanto, estudos recentes sobre o mecanismo antibacteriano da própolis não são claros, porém descrevem maior efetividade quando associados a outros materiais biológicos no tratamento de UC, como o uso de antibióticos orais^(14)^.

Houve modificação de tecido inviável para viável, ou seja, o tecido necrótico deu lugar ao tecido de granulação, com posterior neo-formação de tecido epitelial incipiente, demonstrando que a capacidade de limpeza do PPV foi efetiva. Isso é explicado por apresentar enzimas que favorecem o processo de cicatrização, sendo a enzima mieloperoxidase uma das mais notáveis. Essa auxilia na quebra e dissolução de tecidos inviáveis, facilitando sua remoção e promovendo um ambiente mais propício para o desenvolvimento de tecidos de granulação^(15)^.

Observou-se, no grupo experimental, contínuo seguimento de tecidos de cicatrização durante o tratamento, inclusive naqueles com maior grau de lesão e condições que impossibilitam a microcirculação e a nutrição local. Nutrientes carreados pelo sangue fazem total diferença no desenvolvimento de tecido viável. Indivíduos com dificuldades circulatórias, diabetes *mellitus* e outras condições podem ser prejudicados com a falta de nutrientes para a cicatrização. Portanto, os aminoácidos, vitaminas e carboidratos presentes na própolis favorecem a nutrição local para tecidos das lesões, melhorando o microambiente dos tecidos quando aplicado topicamente^(14)^.

Vale salientar que houve a presença de indivíduos diabéticos e não diabéticos nesta pesquisa em ambos os grupos. A análise de comparação múltipla mostrou que não há efeito estatisticamente significativo para o tipo de tratamento entre os grupos, nem para o fato de ser ou não diabético, bem como para a interação entre essas duas variáveis. Portanto, o fato de conter indivíduos diabéticos na amostra, apesar da condição retardar a cicatrização de feridas, não apresentou diferença entre os participantes e o potencial efeito do tratamento, apontando que a PPV é um elemento cicatrizante para ambos os tipos de feridas.

A redução da dor na UC foi um dos benefícios observados para o grupo experimental, o que pode estar diretamente associado ao poder de debridamento e limpeza da ferida com o uso da PPV. As UC estão relacionadas à inflamação, que é mediada por substâncias químicas como citocinas, prostaglandinas e fatores de crescimento. Essas substâncias irritam os nervos nas proximidades da ferida, desencadeando a percepção da dor. Além disso, a presença de tecido necrótico e exsudato nas feridas pode criar um ambiente ácido e propenso à infecção, piorando a dor. A remoção do tecido inviável e o controle da inflamação são abordagens cruciais para reduzir a dor^(16)^.

Além disso, estudos comprovam que o efeito anti-inflamatório da própolis assemelha-se aos anti-inflamatórios não esteroidais, apresentando ação benéfica no metabolismo da fibronectina ao inibir a biossíntese da fibronectina nativa para reduzir sua degradação no tecido danificado^(17)^. Também foi demonstrado que, em feridas de queimadura tratadas com PPV, a concentração de radicais livres é menor do que em feridas tratadas com sulfadiazina de prata^(18)^.

### Contribuições para a área da enfermagem, saúde ou política pública

Como contribuição para o avanço na prática do cuidado em enfermagem, este estudo apresenta resultados importantes de um recurso natural que exprime efetividade e pode ser utilizado na assistência de enfermagem para o tratamento de UC. Com o avanço dos recursos tecnológicos, inúmeros produtos são desenvolvidos e testados para o tratamento de UC; entretanto, sabe-se que o custo-benefício também é um fator que deve ser avaliado na escolha do produto. Portanto, a própolis verde brasileira é uma opção confiável e comprovada quanto à sua efetividade, pois é de fácil acesso e baixo custo, podendo ser implementada de forma direta na assistência.

Ainda, este estudo abre caminhos para o desenvolvimento de novas pesquisas, utilizando novas técnicas de comparação e apresentando evidências de forma sistematizada, pois a enfermagem é uma ciência que busca implementar em seus cuidados a prática baseada em evidência.

### Limitações do estudo

Podem-se considerar como limitações deste estudo o tamanho da amostra, a duração da pesquisa, a exclusão de pacientes (imunossuprimidos por causas genéticas, transtornos psiquiátricos e/ou em tratamento cicatricial prévio) e o fato de o estudo ser unicêntrico.

## CONCLUSÕES

Esta pesquisa pôde apresentar dados acerca dos efeitos da própolis verde brasileira no tratamento de UC. Seus resultados evidenciam que esse recurso tem potencial cicatrizante e possibilita a degradação do tecido inviável, para o aparecimento do tecido de granulação. Também foram identificadas modificação na característica da borda, redução da dor e diminuição da carga microbiana, possibilitando diferencial progressivo no tipo de tecido encontrado no leito das UC tratadas, como também às de pacientes com condições associadas, como o diabetes *mellitus.*

Quando comparado com a população controle, sugere-se que a própolis possivelmente possui efeito cicatrizante superior ao dos AGE, apresentando mais eficácia no preparo do leito da UC em aspéctos clínicos e macroscópicos, sugerindo ser um produto de limpeza e cicatrização de UC com um efeito debridante.
